# Supplementary material for: The multiple myeloma risk allele at 5q15 lowers ELL2 expression and increases ribosomal gene expression
Source: Nat Commun. 2018 Apr 25;9:1649. doi: 10.1038/s41467-018-04082-2 (PMC5917026; doi:10.1038/s41467-018-04082-2)
Supplement: Supplementary file 1 — Supplementary Information [file 41467_2018_4082_MOESM1_ESM.pdf]

Supplementary Figure 1

a

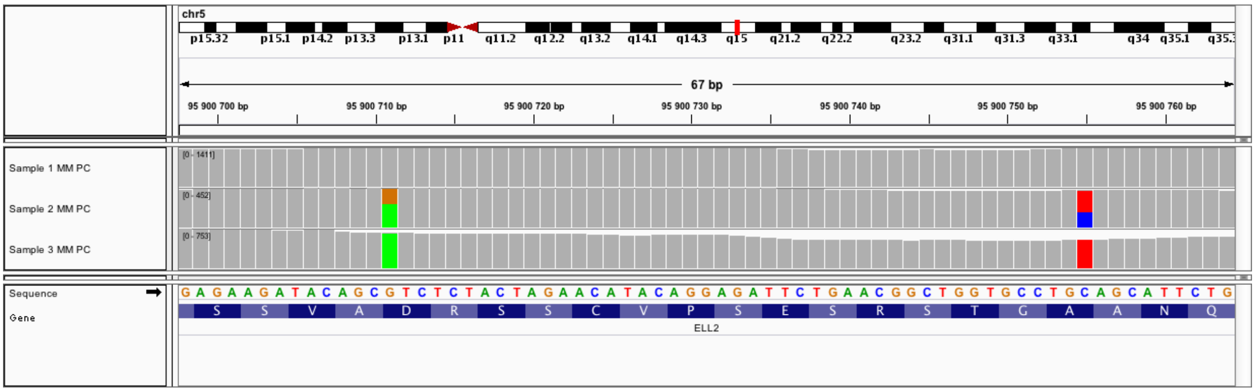

b

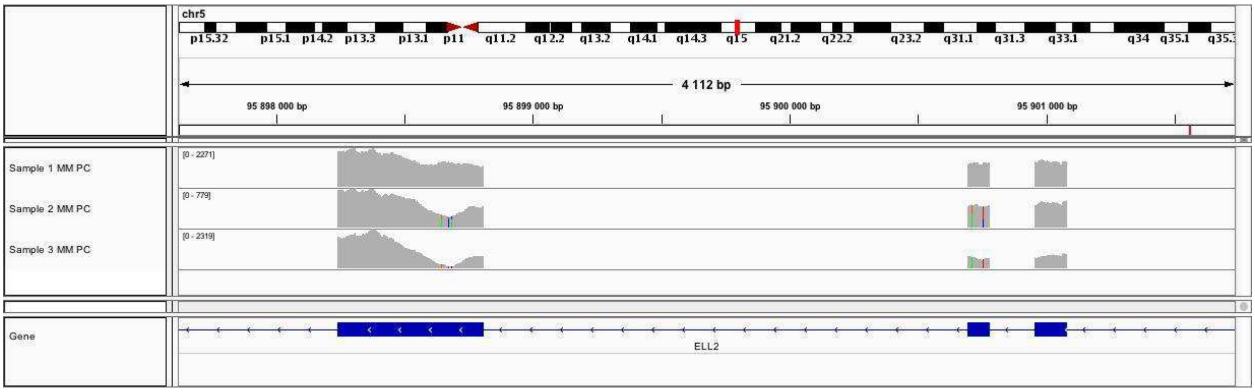

Genotyping. We genotyped the Swedish-Norwegian samples for the *ELL2* MM risk allele using the coding variant rs3815768, which could be detected in the mRNA sequencing data. These screen shots from Integrative Genomics Viewer show aligned reads from an individual homozygous for the risk/reference allele ("Sample 1"), a heterozygous individual ("Sample 2"), and an individual homozygous for the protective/alternative allele ("Sample 3"). **(a)** Close-up of exon 7 showing the two linked, coding variants rs17085249 G > T and rs3815768 C > T. Colors indicate sequence deviation from the reference genome assembly. **(b)** Overview of *ELL2* exons 6, 7 and 8. The linked, coding variants rs3815768, rs17085249 in exon 7, and rs3777202, rs3777203 and rs3777204 in exon 8 create an alignment bias, producing underestimated expression values for exon 6, 7 and 8. This technical artifact explains why the eQTL effects for *ELL2* exons 6, 7 and 8 were less significant (**Table 1**).

Supplementary Figure 2

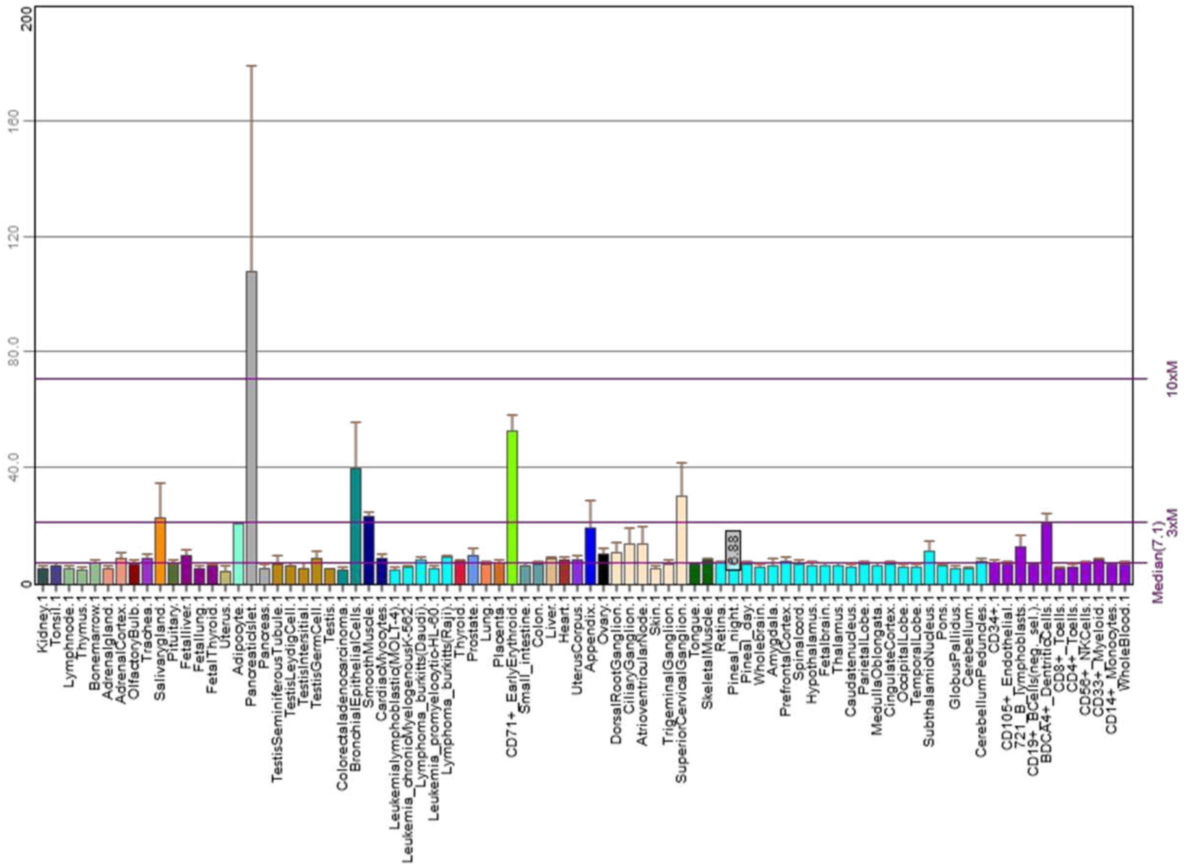

Expression of *ELL2* across human tissues. This plot illustrates *ELL2* expression values in the GeneAtlas data set (NCBI Gene Expression Omnibus accession number GSE1133; Affymetrix U133A probe set 214446_at).

Supplementary Figure 3

a **b**

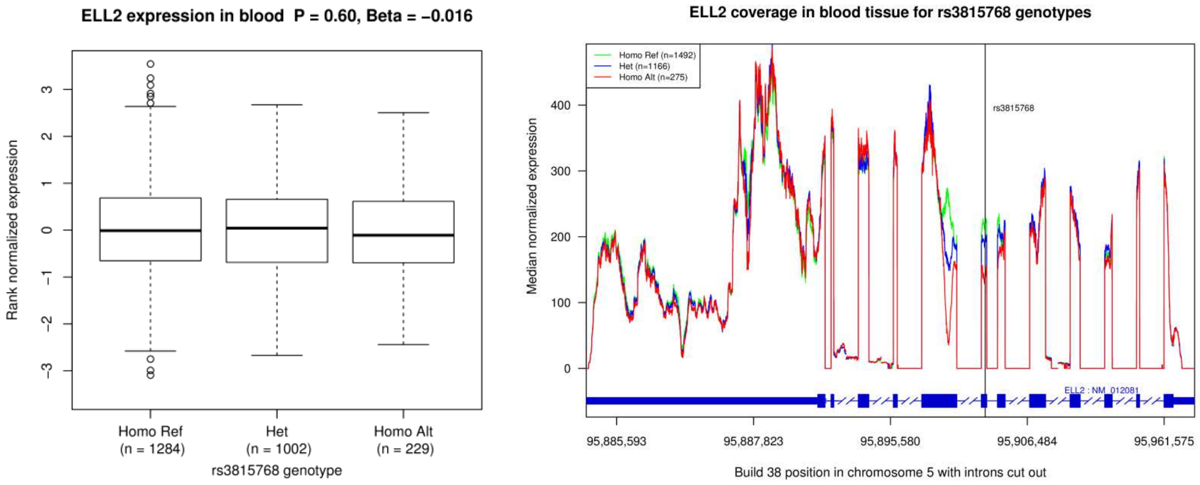

***ELL2* expression *vs* genotype in blood mRNA sequencing data for 2,515 Icelanders.** In contrast to the highly reproducible effect detected in MM plasma cells, we could not detect any effect on *ELL2* expression in these data (**a**) despite good sequence coverage (**b**).

Supplementary Figure 4

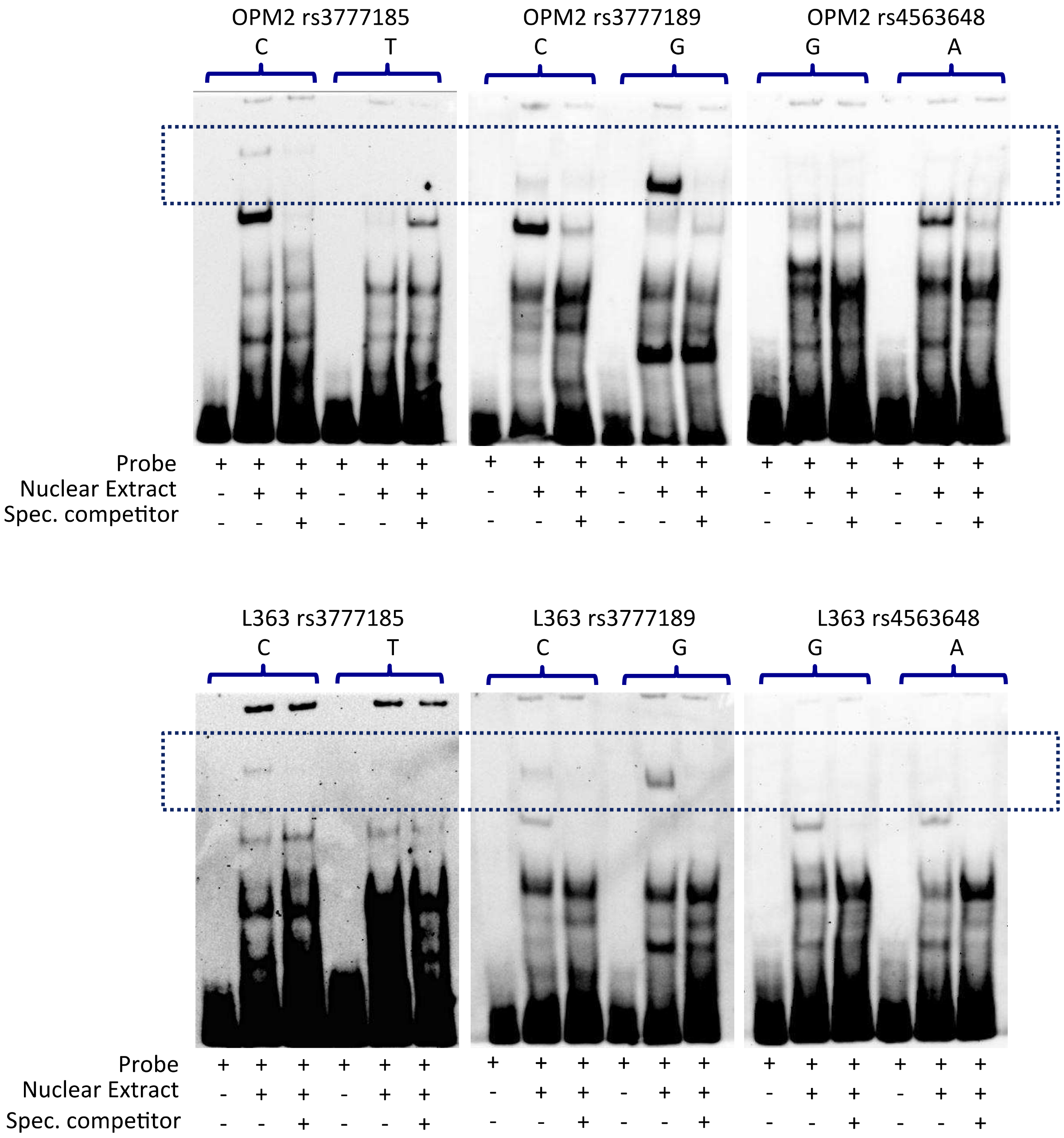

Electrophoretic mobility shift assays for rs3777185, rs3777189 and rs4563648. Nuclear extracts from OPM2 and L363 cells were mixed with biotin-labeled probes representing 25 nt of genomic sequence with either the risk/low-expressing allele or protective/high-expressing in the center. Unlabeled competitor probes with the same sequences were used to test binding specificity. As indicated by the dashed box, we observed allele-dependent binding of nuclear proteins for rs3777185 and rs3777189, whereas the results were less convincing for rs4563648. For rs3777185, we observed loss of one band with the T-allele. For rs3777189, we observed increased intensity of one band with the G allele. As is common in this type of experiments, we also observed several additional bands of variable intensity that could not be convincingly inhibited by unlabeled competitor probes, and thus likely unspecific.

Supplementary Figure 5

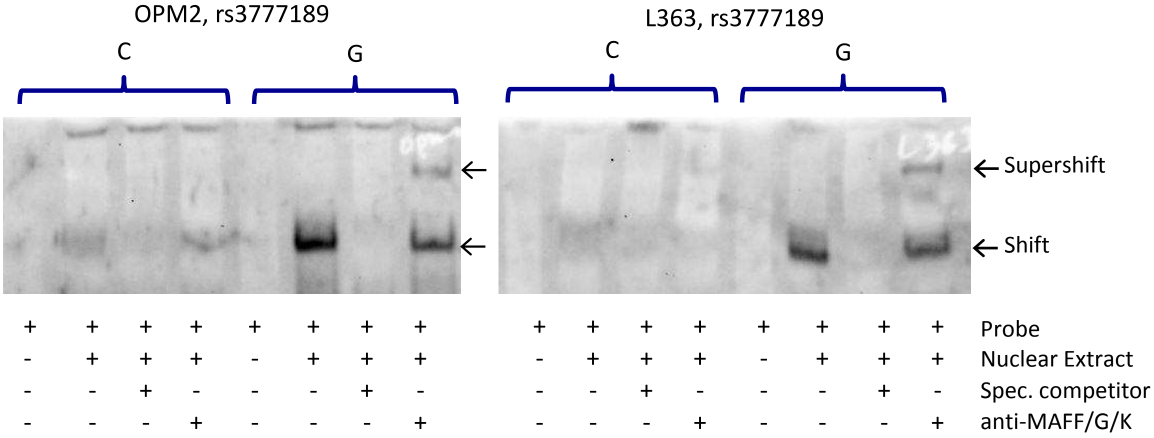

Electrophoretic mobility shift assays for rs3777189. Nuclear extracts from OPM2 and L363 cells were incubated with biotin-labeled probes representing 25 nt of genomic sequencing with either the risk/low-expressing (C) or protective/high-expressing (G) variant in the center. Unlabeled, specific competitor probes with the same sequences were used to assess binding specificity. As shown, incubation with antibody targeting MAFF/G/K yielded an allele-specific supershift for the G-allele, but not for the C-allele. These results, along with **Fig. 2c**, **Fig. 3a** and **Supplementary Table 4** and **5**, identify rs3777189 as a likely casual variant, where the reduced *ELL2* expression associated with the risk allele could be mediated by loss of a stimulatory binding site for at least one of the MAFK/G/K factors.

Supplementary Figure 6

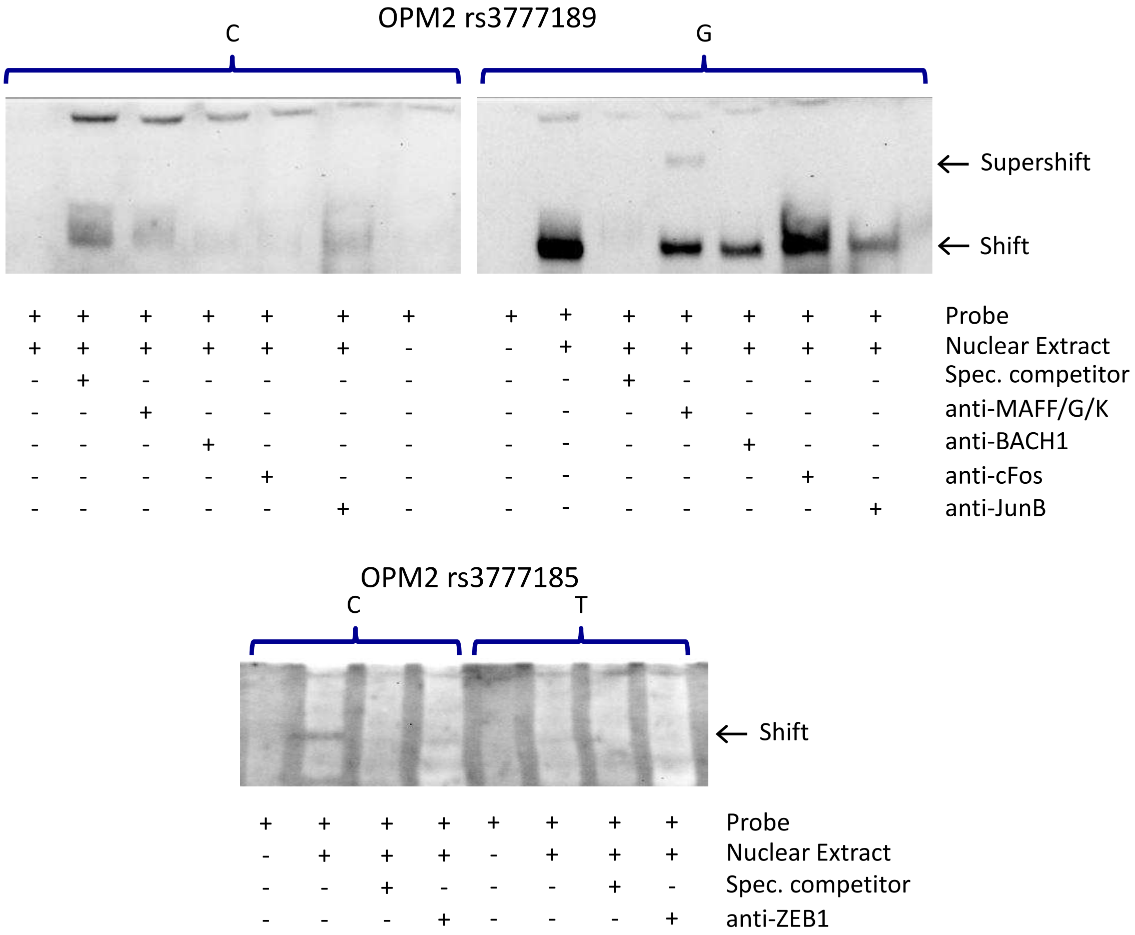

Additional electrophoretic mobility shift assays for rs3777189 and rs3777185. Nuclear extracts from OPM2 cells were incubated with biotin-labeled probes representing 25 nucleotides of genomic sequence with either the risk/low-expressing (C and C) or protective/high-expressing (G and T) variants in the center. Unlabeled, specific competitor probes with identical sequences were used to assess binding specificity. Based on **Supplementary Table 4** and **5**, we tested antibodies towards BACH1, JUNB, c-FOS or MAFF/G/K for rs3777189, and antibodies towards ZEB1 for rs3777185. Out of the tested antibodies, only the MAFF/G/K antibody yielded an allele-specific supershift.

Supplementary Figure 7

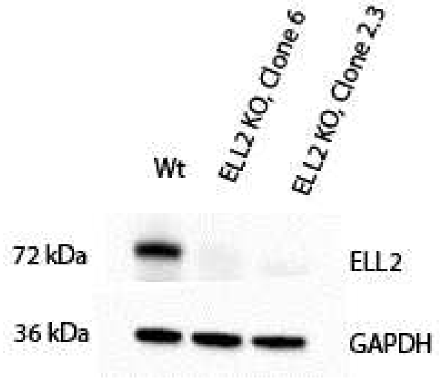

Western blots in ELL2-knockout cells. Verification of successful *ELL2* CRISPR-Cas9 knockout in two L363 clones using Western blot with antibodies towards ELL2.

Supplementary Figure 8

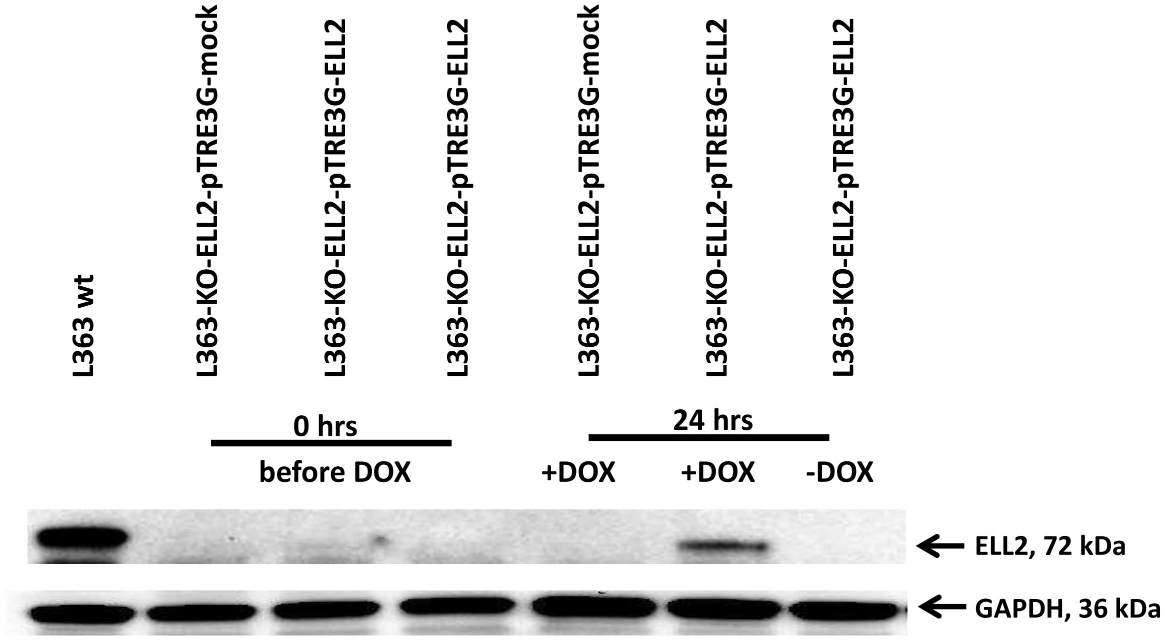

Reconstitution of ELL2 expression in L363-knockout cells. This figure shows Western blots of wildtype L363 cells (lane 1), L363-*ELL2*-KO cells transfected with mock vector (lanes 2 and 5) or CRISPR-resistant *ELL2* controlled by a doxycycline (DOX)-dependent promoter (lanes 3-4 and 6-7). The cell cultured were samples at 0 hours (*i.e.*, before addition of DOX) and at 24 hours. As shown, we achieved successful reconstitution of *ELL2* expression at 24 hours in the *ELL2*-transfected L363-KO cells cultured with DOX.

Supplementary Table 1

Variants in LD (r^2>0.8) with rs9314162 retrieved from HaploReg 4.1. Variants selected for functional evaluation indicated in grey.

pos (hg38)	LD (r^2)	LD (D')	Variant (rsID)	Ref	Alt	AFR freq	AMR freq	ASN freq	EUR freq	Promoter histone marks	Enhancer histone marks	DNAse	Proteins bound	Motifs changed	dbSNP func annot
5:95883557	0.81	-0.98	rs147295	T	C	0.36	0.66	0.64	0.67		ESDR, LNG, FAT, STRM, BRST, BLD, MUS, LIV, PANC, PLCNT, GI, CRVX, SKIN, BONE	BLD,BLD,MUS, BLD,CRVX		Pbx3,Sox	
5:95885633	0.95	0.97	rs1043381	C	T	0.55	0.31	0.36	0.29					Pbx-1	3'-UTR
5:95886452	0.96	0.98	rs17085231	C	T	0.46	0.24	0.32	0.29		LIV			Irf	3'-UTR
5:95886807	0.96	0.98	rs11135442	T	C	0.50	0.24	0.32	0.29		LIV				3'-UTR
5:95890951	0.96	0.98	rs6556893	A	G	0.50	0.24	0.32	0.29		LNG, LIV			CEBPG,DMRT4,DMRT7,ZEB1	intronic
5:95891758	0.95	0.97	rs6879652	T	C	0.64	0.31	0.36	0.29		LNG, BLD, LIV, BRN, PANC, PLCNT	BLD,SKIN		HNF4,Hsf,NF-I	intronic
5:95891803	0.96	0.98	rs10035477	T	C	0.25	0.23	0.32	0.29			BLD,SKIN,HRT, MUS,BLD		AP-2,EWSR1-FLI1	intronic
5:95893132	0.92	0.97	rs3777208	A	G	0.49	0.24	0.32	0.29		LIV	SKIN	CTCF	PEBP,Pou1f1,Sox	intronic
5:95893725	0.96	0.98	rs6890954	T	C	0.51	0.24	0.32	0.29					PTF1-beta	intronic
5:95896837	0.96	0.98	rs2546191	G	A	0.64	0.31	0.47	0.29		ESC, ESDR, IPSC, FAT, STRM, BRN, GI, PANC, BLD, MUS			GR,Nkx2	intronic
5:95896847	0.96	0.98	rs967546	G	A	0.45	0.24	0.43	0.29		ESC, ESDR, IPSC, FAT, STRM, BRN, GI, PANC, BLD, MUS	SKIN		GR	intronic
5:95897400	0.95	0.97	rs3836904	TG	T	0.44	0.24	0.43	0.29		ESC, FAT, STRM, GI, PANC, LIV	BRN		Mef2,Nkx2,STAT	intronic
5:95898646	0.96	0.98	rs3777204	A	G	0.61	0.31	0.47	0.29		FAT, ESC, GI, PANC			CAC-binding-protein,CACD,CCNT2,Irf,Klf4,Klf7,MAZR,MZF1::1-4,NF-kappaB,Pax-4,RREB-1,SP1,STAT,TATA,UF1H3BETA,Zfp281	synonymous
5:95898673	0.96	0.98	rs3777203	C	T	0.45	0.24	0.43	0.29		FAT, ESC, GI, PANC			AP-2rep,BCL,BDP1,BHLHE40,CTCF,ELF1,Ets,GR,HEY1,Hic1,NF-E2,Pou2f2,Rad21,TATA,TCF12,WT1,YY1,p300	synonymous
5:95898688	0.96	0.98	rs3777202	A	C	0.52	0.24	0.43	0.29		FAT, ESC, GI, PANC				synonymous
5:95898926	0.96	0.98	rs3777201	G	T	0.46	0.24	0.43	0.29		FAT, GI, PANC, BLD, LIV			Arid5a	intronic
5:95899087	0.96	0.98	rs3777200	C	T	0.46	0.24	0.43	0.29		FAT, GI, PANC, BLD, LIV			Foxa,Foxc1,Foxd1,Foxj2,Foxo,Maf,Nkx2,Nkx3,TCF12,p300	intronic
5:95899489	0.96	0.98	rs2042340	T	C	0.55	0.24	0.43	0.29		FAT, BLD, STRM, LIV, BRN, PANC			Hoxa7	intronic
5:95899923	0.96	0.98	rs1057964	T	C	0.62	0.31	0.47	0.29			MUS		Foxa,HDAC2,Hic1,p300	intronic
5:95900044	0.96	0.98	rs1057963	G	C	0.54	0.24	0.43	0.29			BLD		ATF3,BCL,NF-kappaB,SP2,STAT,Zfp281,Zic	intronic
5:95900306	0.96	0.98	rs2348984	A	C	0.64	0.31	0.47	0.29		LNG, FAT, STRM, BRST, MUS, SKIN, LIV, BRN, HRT, PANC, GI, BLD, CRVX, BONE			E2F,Ik-1	intronic

5:95900711	0.96	0.98	rs17085249	G	A	0.35	0.23	0.43	0.29		FAT, STRM, BRST, MUS, SKIN, LIV, BRN, GI, HRT, PANC, CRVX	BRN,HRT		BCL,NRSF	synonymous
5:95900755	0.96	0.98	rs3815768	C	T	0.35	0.23	0.43	0.29		FAT, STRM, BRST, MUS, SKIN, LIV, BRN, GI, HRT, PANC, CRVX	SKIN,BRN,HRT,BLD,SKIN		Rad21	missense
5:95901336	0.96	0.98	rs7716691	C	T	0.61	0.31	0.47	0.29		FAT, STRM, MUS, BRN, PANC, CRVX, BRST, SKIN			AIRE,HNF6,SRF,YY1,Zfp187	intronic
5:95901559	0.89	0.98	rs10671631	T	TAAC	0.59	0.31	0.47	0.28					Dbx1,Foxo,GR,Gsc,HNF1,Hoxb13,Hoxd8,Mef2,Ncx,Obox3,Otx2,Sox,Zfp105	intronic
5:95901661	0.96	0.98	rs7702674	A	C	0.51	0.24	0.43	0.29		ESC, FAT, STRM, MUS, SKIN, BRN, PANC, CRVX, BRST, VAS	BRST,SKIN,MUS,CRVX,BRST,SKIN	POL24H8	HNF4	intronic
5:95902076	0.96	0.98	rs11738768	C	A	0.35	0.23	0.43	0.29	LNG, BRST, BRN	ESC, ESDR, LNG, FAT, STRM, BLD, MUS, SKIN, BRN, GI, KID, PANC, CRVX, VAS, BONE	LNG,BRST,BLD,SKIN,PANC,MUS,GI,CRVX,BRST,BRN,SKIN		Foxa,Foxc1,Foxd1,Foxf2,Foxk1,HDAC2,Hoxb6,Nkx3,Nkx6-2,SETDB1,TCF12,p300	intronic
5:95902212	0.96	0.98	rs3777197	T	C	0.62	0.31	0.47	0.29	LNG	ESC, ESDR, LNG, STRM, BRST, BLD, MUS, SKIN, BRN, GI, KID, PANC, CRVX, VAS, BONE	BRST,SKIN,SKIN,CRVX,MUS,SKIN		DMRT1,DMRT2,DMRT3,DMRT4,DMRT5,DMRT7,Foxf2,Foxl1,Foxq1,Mef2,Pou4f3	intronic
5:95902456	0.96	0.98	rs3777196	A	C	0.62	0.31	0.47	0.29	LNG	LNG, BRST, BLD, STRM, MUS, SKIN, BRN, GI, KID, PANC, CRVX, VAS, BONE	SKIN,GI,GI,GI, BRN,SKIN,LNG		AFP1,Foxa,Foxj2,Foxl1,HNF1,Hoxc9,Hoxd8,Ncx	intronic
5:95902878	0.96	0.98	rs11739652	C	G	0.35	0.23	0.43	0.29		LNG, BRST, BLD, STRM, MUS, SKIN, BRN, GI			HEN1,HNF4,INSM1,LF-A1,Mxi1,Myf,NF-E2,RXRA,Sin3Ak-20	intronic
5:95904955	0.95	0.98	rs56156268	G	A	0.25	0.23	0.43	0.29		FAT			Foxj2	intronic
5:95905161	0.96	0.98	rs11744881	A	T	0.51	0.25	0.43	0.29		FAT			CTCF,Cphx,Zbtb12	intronic
5:95905292	0.95	0.98	rs11741563	C	T	0.35	0.23	0.43	0.29		FAT			Foxj1,Foxk1,Foxp1,Irx,Pou1f1,Pou2f2,Pou3f3,Pou5f1,TATA	intronic
5:95905423	0.94	0.97	rs11741590	C	T	0.61	0.31	0.47	0.29		FAT			FAC1,GR,Gfi1b	intronic
5:95905498	0.92	0.97	rs202194544	T	TTC	0.60	0.31	0.45	0.29		FAT, MUS, BRN			BCL,HDAC2,Pax-5,TATA,p300	intronic
5:95905518	0.94	0.97	rs10069748	T	C	0.62	0.31	0.47	0.29					Mrg1::Hoxa9	intronic
5:95906164	0.96	0.98	rs10070197	T	C	0.62	0.31	0.47	0.29					BRCA1,SP1,SP2	intronic
5:95907227	0.96	0.98	rs56219066	T	C	0.39	0.23	0.43	0.29		LNG, BRST, BLD, MUS, LIV, BRN, GI, HRT	BLD,BLD,LNG,GI,MUS,SKIN		Eomes,Mtf1,RBP-Jkappa	intronic
5:95908710	0.99	1	rs2548594	C	T	0.61	0.31	0.47	0.29		LNG, IPSC, FAT, BRST, STRM, MUS, SKIN, LIV, BRN, PANC, GI, OVRY, BLD, CRVX	LIV		Arid5a,CDP,Fox,Foxc1,Foxi1,Foxj1,Foxo,Foxq1,Irf,Pou2f2	intronic
5:95909680	0.99	1	rs3777194	C	T	0.62	0.31	0.47	0.29		ESC, ESDR, LNG, IPSC, FAT, STRM, BRST, MUS, LIV, BRN, OVRY, BLD, CRVX, SKIN			CDP	intronic
5:95910430	0.99	1	rs2270554	T	G	0.62	0.31	0.47	0.29		ESDR, LNG, STRM, MUS, LIV, BRN, OVRY			Nanog	intronic
5:95911056	0.99	1	rs3777193	G	A	0.62	0.31	0.47	0.29		LNG, FAT, STRM, MUS, LIV, BRN			Gm397,Hmx,Nanog,Nkx2,TCF12	intronic
5:95911163	1	1	rs3777192	G	A	0.35	0.23	0.43	0.29		LNG, FAT, STRM, MUS, LIV, BRN				intronic

5:95911401	0.97	0.99	rs9986236	A	G	0.58	0.30	0.46	0.29		LNG, FAT, STRM, MUS, LIV, BRN			Zbtb3	intronic
5:95911930	0.99	1	rs17085266	A	C	0.62	0.31	0.47	0.29		LNG, FAT, BLD, STRM, MUS, LIV, BRN, GI, HRT	MUS,GI,SKIN	KAP1	CDP,SP2	intronic
5:95912268	1	1	rs3777190	C	T	0.35	0.23	0.43	0.29		LNG, FAT, STRM, MUS, LIV, BRN, GI	ESDR		Maf,Nrf1	intronic
5:95913072	0.99	1	rs3836901	GGT	G	0.61	0.31	0.47	0.29		ESDR, STRM, SKIN, LIV, BRN			ERalpha-a,HNF4,NR4A	intronic
5:95914569	0.99	1	rs13361341	A	T	0.61	0.31	0.47	0.29		STRM, BRN, PANC			Mef2,TATA,Zfp105	intronic
5:95914774	0.98	0.99	rs13361837	T	G	0.65	0.31	0.47	0.29		STRM, BRN, PANC, HRT			AIRE,Irf,Nanog	intronic
5:95915534	0.99	1	rs6889029	G	A	0.49	0.25	0.43	0.29	BRN	FAT, STRM, BLD, MUS, LIV, BRN, HRT, PANC, LNG				intronic
5:95915642	0.84	0.99	rs1458017	G	A	0.60	0.31	0.47	0.26	BRN	FAT, STRM, BLD, MUS, LIV, BRN, HRT, PANC, LNG			Irf,Rad21	intronic
5:95916207	0.99	1	rs1841010	G	C	0.63	0.31	0.47	0.29	BLD	ESC, ESDR, LNG, FAT, STRM, BLD, MUS, SKIN, VAS, LIV, BRN, GI, HRT, THYM, OVRY, PANC, SPLN	BLD,BLD,BLD, HRT,BLD,BLD	PU1	HNF4,RXR::LXR,RXRA	intronic
5:95916890	1	1	rs9314162	C	A	0.27	0.23	0.43	0.29	BLD	ESC, ESDR, LNG, IPSC, FAT, STRM, BRST, BLD, MUS, LIV, BRN, PANC, HRT, GI	ESDR,LIV,MUS	FOXA1,FOXA2	CEBPB,p300	intronic
5:95917404	1	1	rs3777189	C	G	0.53	0.24	0.43	0.29		ESC, ESDR, IPSC, FAT, STRM, BRST, BLD, MUS, LIV, BRN, PANC	BLD,BLD	FOXA1,MAFK	AP-1,AP-2,BAF155,BATF,Bach1,Bach2,GR,HMGN3,KAP1,Maf,Myc,NF-E2,Nrf-2,TCF4,Zfp691,p300	intronic
5:95917591	0.99	1	rs1458018	G	T	0.63	0.31	0.47	0.29		ESC, ESDR, IPSC, FAT, BRST, BLD, STRM, MUS, LIV, BRN, PANC, BONE	SKIN		Foxk1,Irf,NF-I,Nanog,Sox	intronic
5:95917785	1	1	rs1458019	A	G	0.41	0.24	0.43	0.29		ESDR, ESC, FAT, BRST, BLD, STRM, MUS, LIV, BRN, PANC, BONE			CEBPB,Dobox4,Osf2,PEBP,TATA	intronic
5:95918467	0.99	1	rs2547997	G	T	0.61	0.31	0.47	0.29		ESDR, FAT, BRST, BLD, STRM, MUS, LIV, BRN, PANC	BLD		Dobox4,Myc,Sox,TBX5	intronic
5:95918798	1	1	rs3777188	T	C	0.37	0.23	0.43	0.29	BLD	ESDR, LNG, FAT, BRST, BLD, STRM, MUS, BRN, SKIN, LIV, PANC, GI	SKIN,BLD	GATA1	Foxa,Foxd1,Foxj1,Foxk1,Foxo,Obox6,Sox	intronic
5:95919675	0.99	1	rs4642392	G	A	0.64	0.31	0.47	0.29	BLD	ESDR, FAT, BLD, STRM, LIV, BRN, PANC	SKIN			intronic
5:95920020	1	1	rs1423269	A	G	0.40	0.24	0.43	0.29		FAT, BLD, LIV, BRN			Irf,Pou2f2	intronic
	0.99	1	rs147935914	A	ACT	0.61	0.31	0.47	0.29		ESDR, LNG, FAT, BRST, BLD, SKIN, VAS, LIV, BRN, GI, ADRL, HRT, PANC, SPLN			Ets,Maf,Mef2,NF-E2,Smad	intronic
5:95921270	0.99	1	rs1423268	T	C	0.64	0.32	0.47	0.29		LNG, FAT, BRST, BLD, SKIN, VAS, LIV, BRN, GI, HRT, PANC				intronic
5:95922743	1	1	rs2829	T	C	0.35	0.23	0.43	0.29		LNG, FAT, BLD, STRM, MUS, SKIN, LIV, GI			Foxa,GATA,GR,Ik-2,NF-AT	intronic

5:95923050	0.99	1	rs6885567	T	G	0.61	0.31	0.47	0.29		LNG, FAT, BRST, BLD, STRM, MUS, SKIN, LIV, BRN, GI, HRT, PANC			Lhx3,Pou6f1,STAT,TATA	intronic
5:95924158	1	1	rs3777185	C	T	0.51	0.24	0.43	0.29	BLD	ESC, ESDR, LNG, FAT, STRM, BRST, BLD, MUS, SKIN, LIV, BRN, GI, PANC, THYM, HRT, BONE	BLD,BLD,BLD, BLD,BLD	GATA1,POL2	ZEB1	intronic
5:95925834	0.99	1	rs6877329	C	G	0.61	0.31	0.47	0.29	BLD	ESDR, LNG, FAT, STRM, BRST, BLD, SKIN, LIV, BRN, GI, PANC, THYM, BONE			Ets,STAT	intronic
5:95926340	1	1	rs3777184	G	C	0.35	0.23	0.43	0.29	BLD	BLD, STRM, SKIN, LIV, BRN, PANC			GR,HNF4	intronic
5:95928031	0.94	0.97	rs3777183	G	A	0.36	0.23	0.43	0.29		STRM, BLD, SKIN, LIV, BRN, GI, HRT, PANC, MUS	ESDR,BRST,BLD,MUS,BLD,BLD	NFKB,BATF,BCL11A,BCL3,EBF1,IRF4,JUND,MEF2A,MEF2C,P300,RXRA,SP1,STAT3,TBP,TCF12	GR,Myf,Nkx3,SEF-1	intronic
5:95928048	0.94	0.97	rs3777182	T	A	0.36	0.23	0.43	0.29		STRM, BLD, SKIN, LIV, BRN, GI, HRT, PANC, MUS	ESDR,BRST,BLD,BLD,BLD,MUS,BLD,MUS,BLD	NFKB,BATF,BCL11A,BCL3,EBF1,IRF4,JUND,MEF2A,MEF2C,P300,RXRA,SP1,STAT3,TBP,TCF12	FAC1,RREB-1,SEF-1	intronic
5:95928948	0.94	0.97	rs889302	A	C	0.61	0.31	0.47	0.29	BLD	ESDR, ESC, LNG, FAT, STRM, BRST, BLD, SKIN, LIV, GI, THYM, OVRY, PANC, HRT, MUS, CRVX	LNG,GI,LNG,GI,BLD			intronic
5:95929383	0.94	0.97	rs2015159	C	T	0.60	0.32	0.45	0.29		FAT, STRM, BLD, THYM, LIV			BDP1,GR,LUN-1	intronic
5:95929852	0.94	0.97	rs4563648	G	A	0.64	0.31	0.47	0.29	BLD	FAT, STRM, BRN, THYM, BLD, LIV	BLD,THYM,BLD	MEF2A,MEF2C,NFKB,TCF12	Eomes	intronic

Supplementary Table 2

Pairwise r2 and D' values for all variants discussed in the study.

SNP1	SNP2	r2	D'
rs1423269	rs3777189	1	1
rs1423269	rs9314162	1	1
rs1423269	rs3777185	1	1
rs1423269	rs3815768	1	1
rs1423269	rs17085249	1	1
rs1423269	rs1841010	0.957	1
rs1423269	rs6877329	0.957	1
rs1423269	rs56219066	0.957	1
rs1423269	rs3777204	0.957	1
rs1423269	rs3777184	0.956	1
rs1423269	rs4563648	0.913	1
rs1423269	rs889302	0.912	0.955
rs56219066	rs3777204	1	1
rs56219066	rs1841010	1	1
rs56219066	rs6877329	1	1
rs56219066	rs3815768	0.957	1
rs56219066	rs17085249	0.957	1
rs56219066	rs9314162	0.957	1
rs56219066	rs3777189	0.957	1
rs56219066	rs1423269	0.957	1
rs56219066	rs3777185	0.957	1
rs56219066	rs889302	0.957	1
rs56219066	rs3777184	0.915	1
rs56219066	rs4563648	0.874	1
rs9314162	rs3777189	1	1
rs9314162	rs1423269	1	1
rs9314162	rs3777185	1	1
rs9314162	rs3815768	1	1
rs9314162	rs17085249	1	1
rs9314162	rs1841010	0.957	1
rs9314162	rs6877329	0.957	1
rs9314162	rs56219066	0.957	1
rs9314162	rs3777204	0.957	1
rs9314162	rs3777184	0.956	1
rs9314162	rs4563648	0.913	1
rs9314162	rs889302	0.912	0.955
rs1841010	rs56219066	1	1
rs1841010	rs6877329	1	1
rs1841010	rs3777204	1	1
rs1841010	rs9314162	0.957	1
rs1841010	rs3777189	0.957	1
rs1841010	rs1423269	0.957	1
rs1841010	rs3777185	0.957	1
rs1841010	rs889302	0.957	1

rs1841010	rs3815768	0.957	1
rs1841010	rs17085249	0.957	1
rs1841010	rs3777184	0.915	1
rs1841010	rs4563648	0.874	1
rs3815768	rs17085249	1	1
rs3815768	rs9314162	1	1
rs3815768	rs3777189	1	1
rs3815768	rs1423269	1	1
rs3815768	rs3777185	1	1
rs3815768	rs3777204	0.957	1
rs3815768	rs56219066	0.957	1
rs3815768	rs1841010	0.957	1
rs3815768	rs6877329	0.957	1
rs3815768	rs3777184	0.956	1
rs3815768	rs4563648	0.913	1
rs3815768	rs889302	0.912	0.955
rs3777189	rs9314162	1	1
rs3777189	rs1423269	1	1
rs3777189	rs3777185	1	1
rs3777189	rs3815768	1	1
rs3777189	rs17085249	1	1
rs3777189	rs1841010	0.957	1
rs3777189	rs6877329	0.957	1
rs3777189	rs56219066	0.957	1
rs3777189	rs3777204	0.957	1
rs3777189	rs3777184	0.956	1
rs3777189	rs4563648	0.913	1
rs3777189	rs889302	0.912	0.955
rs3777185	rs1423269	1	1
rs3777185	rs3777189	1	1
rs3777185	rs9314162	1	1
rs3777185	rs3815768	1	1
rs3777185	rs17085249	1	1
rs3777185	rs6877329	0.957	1
rs3777185	rs1841010	0.957	1
rs3777185	rs56219066	0.957	1
rs3777185	rs3777204	0.957	1
rs3777185	rs3777184	0.956	1
rs3777185	rs4563648	0.913	1
rs3777185	rs889302	0.912	0.955
rs4563648	rs889302	0.913	1
rs4563648	rs3777185	0.913	1
rs4563648	rs1423269	0.913	1
rs4563648	rs3777189	0.913	1
rs4563648	rs9314162	0.913	1
rs4563648	rs3815768	0.913	1
rs4563648	rs17085249	0.913	1
rs4563648	rs6877329	0.874	1
rs4563648	rs1841010	0.874	1
rs4563648	rs56219066	0.874	1

rs4563648	rs3777204	0.874	1
rs4563648	rs3777184	0.866	0.952
rs6877329	rs1841010	1	1
rs6877329	rs56219066	1	1
rs6877329	rs3777204	1	1
rs6877329	rs3777185	0.957	1
rs6877329	rs889302	0.957	1
rs6877329	rs1423269	0.957	1
rs6877329	rs3777189	0.957	1
rs6877329	rs9314162	0.957	1
rs6877329	rs3815768	0.957	1
rs6877329	rs17085249	0.957	1
rs6877329	rs3777184	0.915	1
rs6877329	rs4563648	0.874	1
rs3777184	rs3777185	0.956	1
rs3777184	rs1423269	0.956	1
rs3777184	rs3777189	0.956	1
rs3777184	rs9314162	0.956	1
rs3777184	rs3815768	0.956	1
rs3777184	rs17085249	0.956	1
rs3777184	rs6877329	0.915	1
rs3777184	rs1841010	0.915	1
rs3777184	rs56219066	0.915	1
rs3777184	rs3777204	0.915	1
rs3777184	rs889302	0.869	0.953
rs3777184	rs4563648	0.866	0.952
rs889302	rs6877329	0.957	1
rs889302	rs1841010	0.957	1
rs889302	rs56219066	0.957	1
rs889302	rs3777204	0.957	1
rs889302	rs4563648	0.913	1
rs889302	rs3777185	0.912	0.955
rs889302	rs1423269	0.912	0.955
rs889302	rs3777189	0.912	0.955
rs889302	rs9314162	0.912	0.955
rs889302	rs3815768	0.912	0.955
rs889302	rs17085249	0.912	0.955
rs889302	rs3777184	0.869	0.953
rs3777204	rs56219066	1	1
rs3777204	rs1841010	1	1
rs3777204	rs6877329	1	1
rs3777204	rs17085249	0.957	1
rs3777204	rs3815768	0.957	1
rs3777204	rs9314162	0.957	1
rs3777204	rs3777189	0.957	1
rs3777204	rs1423269	0.957	1
rs3777204	rs3777185	0.957	1
rs3777204	rs889302	0.957	1
rs3777204	rs3777184	0.915	1
rs3777204	rs4563648	0.874	1

rs17085249	rs3815768	1	1
rs17085249	rs9314162	1	1
rs17085249	rs3777189	1	1
rs17085249	rs1423269	1	1
rs17085249	rs3777185	1	1
rs17085249	rs3777204	0.957	1
rs17085249	rs56219066	0.957	1
rs17085249	rs1841010	0.957	1
rs17085249	rs6877329	0.957	1
rs17085249	rs3777184	0.956	1
rs17085249	rs4563648	0.913	1
rs17085249	rs889302	0.912	0.955

Supplementary Table 3

Reference and alternative allele sequences surrounding the five variants in *ELL2* selected for functional evaluation using a luciferase assays. The sequences were selected from the negative strand due to the orientation of the ELL2 gene.

SNP	Chr	Bp (hg38)	Ref	Alt	Sequence
rs1841010	5	95916207	G	C	CTTGTGTTCAGTGTTTCAGTACATAGCAGCTTCCATATACCTCGTGACCTGGGCATGAT[C/G]TTTAACTTTTTTCCCCCACCTCTCTCTGAGTGTGGCTAACTACTGACCACGTTTTGTCAG
rs9314162	5	95916890	C	A	AGGGGCAGTATGTTCAGCTTATTCTCACTATGTAAATTACTTAGTAAATAATAGGAAGA[G/T]ATGTTGAAATACAAACTTTCTGCCACCAGACCTTCACTCTATTGCAGTCATTTTCTCCCA
rs3777189	5	95917404	C	G	CATGCTCTTCAAGGTCAGAGGCTTTCTTATGCCCTTTTTCTAATTTCACAGTGCTGACT[G/C]AGCTCAAAATACATGCTTATTGACTCAACCTGGAGATAAAAGGGTAAAAACAGATGCTCA
rs3777185	5	95924158	C	T	AATAGGCTCCTGCATATCAGAGGGGACTGACGCATATCCACAGGTTCCTCTGAAACTCT[G/A]CCTGAATGGCTCTAGATTAGACAAGGTCTCAGACCCACCCTAGCTCTGGCCTTCAACAGT
rs6877329	5	95925834	C	G	ATAGTCAAATAATAGATTTTAGATAAAATTAAGAGATGGACTG[G/C]CTTCCTGGGATGCCGGAGGGGAGGTGGGTTCTGAGGCCATACAAATTGGGAAACTATTCAGTATGAAAAGACCCAC
rs3777184	5	95926340	G	C	TCAGTAAAATAGCTATCATATGGAAATTTCAGACGGCTCTAATATGTGTTGGATCCTCT[C/G]TACCCTCTGCCCTTCCCTGTCAGTAGGAATATTTAGCTCTTATTGATTTCTCTAATACTT
rs889302	5	95928948	A	C	ACAGAGTAGCGTGTTCCGGCACCCAGCAAGCACGGTATTGCTGCTGGACATGAAATACAA[T/G]GTGGAGATGAGGCCCAGTTAAACTGGTTTAGTAGCAAGAGTGCTGACAAGGAAACATAT
rs4563648	5	95929852	G	A	GCCACTTGGCCTAGGCTTTGGGGCTAAACATAGCATAAGAGAGGTTAGAAACTTTCTCA[C/T]CCTGACATTTGTACCACACAGCAGGCAGCTGTGTGGGGGTTTTTTTGTTTTTTGTTGTTT

Supplementary Table 4

Transcription factor motifs predicted to be gained or lost by rs3777189, rs3777185, and rs4563648. For this analysis, we used tool PERFECTOS-APE (http://opera.autosome.ru/perfectosape) with the HOCOMOCO-10, JASPAR, HT-SELEX, SwissRegulon and HOMER motif data bases and default filtering parameters (P-value 1 or P-value 2 < 0.0005; fold change >5).

		Risk allele			Protective allele					
**Variant**	**motif**	**strand**	**sequence**	**P-value**	**strand**	**sequence**	**P-value**	**fold**	**database**	**effect of risk variant**
rs3777189	BATF_HUMAN.H10MO.S	pos	ctCagtcagc	2,82E-04	pos	ctGagtcagc	2,05E-05	13,74	HOCOMOCO	loss
rs3777189	SMRC1_HUMAN.H10MO.D	pos	agctCagtcag	2,56E-04	pos	agctGagtcag	1,17E-05	21,91	HOCOMOCO	loss
rs3777189	MAFG_HUMAN.H10MO.C	neg	cacagtgctgactGagctcaa	8,41E-06	neg	cacagtgctgactCagctcaa	3,20E-07	26,28	HOCOMOCO	loss
rs3777189	NF2L2_HUMAN.H10MO.D	pos	gctCagtcagca	2,01E-04	pos	gctGagtcagca	1,58E-05	12,70	HOCOMOCO	loss
rs3777189	FOSL2_HUMAN.H10MO.A	neg	ctgactGagct	6,79E-04	neg	ctgactCagct	2,84E-05	23,94	HOCOMOCO	loss
rs3777189	JUNB_HUMAN.H10MO.C	pos	agctCagtcag	6,16E-04	pos	agctGagtcag	2,84E-05	21,71	HOCOMOCO	loss
rs3777189	JUND_HUMAN.H10MO.A	pos	gctCagtcagc	3,27E-04	pos	gctGagtcagc	1,81E-05	18,03	HOCOMOCO	loss
rs3777189	NRL_HUMAN.H10MO.D	neg	tgctgactGag	1,17E-05	neg	tgctgactCag	5,84E-07	20,00	HOCOMOCO	loss
rs3777189	FOSB_HUMAN.H10MO.C	neg	gctgactGagc	3,60E-04	neg	gctgactCagc	1,57E-05	22,88	HOCOMOCO	loss
rs3777189	MAFK_HUMAN.H10MO.A	neg	cacagtgctgactGagctcaaa	3,50E-06	neg	cacagtgctgactCagctcaaa	4,73E-07	7,39	HOCOMOCO	loss
rs3777189	MAFK_HUMAN.H10MO.S	neg	tgctgactGa	9,79E-05	neg	tgctgactCa	2,34E-06	41,89	HOCOMOCO	loss
rs3777189	FOS_HUMAN.H10MO.A	pos	ctCagtcagca	9,10E-04	pos	ctGagtcagca	2,98E-05	30,56	HOCOMOCO	loss
rs3777189	NFE2_HUMAN.H10MO.B	pos	gagctCagtcagca	2,69E-04	pos	gagctGagtcagca	2,71E-05	9,91	HOCOMOCO	loss
rs3777189	FOSL1_HUMAN.H10MO.A	neg	gctgactGagc	7,86E-04	neg	gctgactCagc	2,44E-05	32,18	HOCOMOCO	loss
rs3777189	ZSC16_HUMAN.H10MO.D	pos	tttgagctCagtcagcactg	2,82E-04	pos	tttgagctGagtcagcactg	0,0026635	0,11	HOCOMOCO	gain
rs3777189	MAFF_HUMAN.H10MO.A	neg	cacagtgctgactGagctcaaa	3,33E-06	neg	cacagtgctgactCagctcaaa	5,21E-07	6,38	HOCOMOCO	loss
rs3777189	JUN_HUMAN.H10MO.A	pos	ctCagtcag	4,58E-04	pos	ctGagtcag	2,85E-05	16,03	HOCOMOCO	loss
rs3777189	BACH1_HUMAN.H10MO.A	neg	tgctgactGagct	1,06E-04	neg	tgctgactCagct	4,25E-06	25,05	HOCOMOCO	loss
rs3777189	MAFA_HUMAN.H10MO.D	neg	gtgctgactGagctcaaaata	0,0014122	neg	gtgctgactCagctcaaaata	2,44E-04	5,79	HOCOMOCO	loss
rs3777189	BATF_HUMAN.H10MO.A	pos	tattttgagctCagtcag	6,16E-04	pos	tattttgagctGagtcag	2,46E-05	25,04	HOCOMOCO	loss
rs3777185	NFIC_HUMAN.H10MO.A	pos	ttcaggCagagtttcag	1,82E-04	pos	ttcaggTagagtttcag	0,0039352	0,05	HOCOMOCO	gain
rs4563648	TBX20_HUMAN.H10MO.D	pos	aaatgtcaggGtgagaa	2,46E-05	pos	aaatgtcaggAtgagaa	1,65E-04	0,15	HOCOMOCO	gain
rs4563648	NR1H2_HUMAN.H10MO.D	pos	tgtggtacaaatgtcaggG	1,36E-04	pos	tgtggtacaaatgtcaggA	7,13E-04	0,19	HOCOMOCO	gain
rs4563648	INSM1_HUMAN.H10MO.C	pos	tgtcaggGtgag	7,53E-05	pos	tgtcaggAtgag	0,0010535	0,07	HOCOMOCO	gain
rs4563648	SOX2_HUMAN.H10MO.B	neg	actttctcaCcctgac	0,0012808	neg	actttctcaTcctgac	1,50E-04	8,55	HOCOMOCO	loss
rs4563648	ETS1_HUMAN.H10MO.C	pos	tcaggGtga	0,0120852	pos	tcaggAtga	4,79E-04	25,25	HOCOMOCO	loss
rs4563648	TBR1_HUMAN.H10MO.D	pos	caggGtgagaaag	1,82E-04	pos	caggAtgagaaag	0,0120841	0,02	HOCOMOCO	gain
rs4563648	TBX21_HUMAN.H10MO.D	pos	aggGtgagaaag	3,97E-04	pos	aggAtgagaaag	0,0067308	0,06	HOCOMOCO	gain
rs4563648	IRF8_HUMAN.H10MO.D	pos	caggGtgagaaagtt	0,0024157	pos	caggAtgagaaagtt	2,32E-04	10,40	HOCOMOCO	loss

rs4563648	KLF8_HUMAN.H10MO.C	pos	caggGtgag	2,94E-04	pos	caggAtgag	0,0047473	0,06	HOCOMOCO	gain
rs4563648	IRF4_HUMAN.H10MO.C	pos	tgtcaggGtgagaaag	0,0024155	pos	tgtcaggAtgagaaag	2,11E-04	11,47	HOCOMOCO	loss
rs4563648	VDR_HUMAN.H10MO.B	pos	aggGtgagaaagtttc	8,75E-05	pos	aggAtgagaaagtttc	0,001483	0,06	HOCOMOCO	gain
rs4563648	BC11A_HUMAN.H10MO.C	pos	atgtcaggGtgagaaag	0,0027959	pos	atgtcaggAtgagaaag	2,82E-04	9,91	HOCOMOCO	loss
rs4563648	NR6A1_HUMAN.H10MO.B	pos	aatgtcaggGtga	4,17E-04	pos	aatgtcaggAtga	0,0024155	0,17	HOCOMOCO	gain
rs3777189	MA0150.2 Nfe2l2	neg	agtgctgactGagct	2,82E-04	pos	tgagctGagtcagca	2,99E-05	9,44	JASPAR	loss
rs3777189	MA0477.1 FOSL1	neg	gctgactGagc	7,84E-04	neg	gctgactCagc	2,71E-05	28,97	JASPAR	loss
rs3777189	MA0476.1 FOS	neg	gctgactGagc	0,0014819	neg	gctgactCagc	5,90E-05	25,12	JASPAR	loss
rs3777189	MA0462.1 BATF+JUN	neg	gtgctgactGa	1,91E-04	pos	gagctGagtca	2,01E-05	9,49	JASPAR	loss
rs3777189	MA0478.1 FOSL2	neg	tgctgactGag	0,0018018	neg	tgctgactCag	7,54E-05	23,88	JASPAR	loss
rs3777189	MA0489.1 JUN	neg	cagtgctgactGag	0,0015572	pos	ttgagctGagtcag	5,37E-05	28,98	JASPAR	loss
rs3777189	MA0491.1 JUND	neg	gctgactGagc	0,0011612	neg	gctgactCagc	5,35E-05	21,70	JASPAR	loss
rs3777189	MA0501.1 NFE2+MAF	neg	ctgactGagctcaaa	2,01E-04	pos	ctGagtcagcactgt	1,18E-05	16,94	JASPAR	loss
rs3777189	MA0490.1 JUNB	neg	tgctgactGag	0,001218	neg	tgctgactCag	5,62E-05	21,65	JASPAR	loss
rs3777189	MA0591.1 Bach1+Mafk	neg	gtgctgactGagctc	4,64E-05	pos	gagctGagtcagcac	1,14E-06	40,84	JASPAR	loss
rs3777185	MA0103.2 ZEB1	neg	ctctGcctg	0,0041068	neg	ctctAcctg	2,19E-04	18,73	JASPAR	loss
rs4563648	MA0517.1 STAT2+STAT1	neg	aaactttctcaCcct	0,0035687	neg	aaactttctcaTcct	2,96E-04	12,04	JASPAR	loss
rs4563648	MA0599.1 KLF5	neg	ttctcaCcct	3,78E-04	neg	ttctcaTcct	0,0064101	0,06	JASPAR	gain
rs4563648	MA0142.1 Pou5f1+Sox2	neg	ctttctcaCcctgac	0,0041314	neg	ctttctcaTcctgac	1,65E-04	25,04	JASPAR	loss
rs4563648	MA0155.1 INSM1	pos	tgtcaggGtgag	9,19E-05	pos	tgtcaggAtgag	0,001634	0,06	JASPAR	gain
rs3777189	MAFK_2	neg	acagtgctgactGagctcaaa	4,46E-06	neg	acagtgctgactCagctcaaa	1,54E-07	28,97	HT-SELEX	loss
rs3777189	ZNF435	neg	agtgctgactGagctcaa	1,73E-04	neg	agtgctgactCagctcaa	0,0012811	0,14	HT-SELEX	gain
rs3777189	MAFG	neg	acagtgctgactGagctcaaa	4,92E-06	neg	acagtgctgactCagctcaaa	1,96E-07	25,04	HT-SELEX	loss
rs3777189	JDP2_5	neg	ctgactGag	0,0051747	neg	ctgactCag	4,79E-04	10,81	HT-SELEX	loss
rs3777189	MAFK_3	pos	ttttgagctCag	0,0115122	pos	ttttgagctGag	4,83E-04	23,85	HT-SELEX	loss
rs3777189	MAFK_4	pos	gagctCagtcagcac	1,37E-05	neg	gtgctgactCagctc	8,91E-07	15,38	HT-SELEX	loss
rs3777189	MAFF	neg	gtgctgactGagctc	2,23E-05	neg	gtgctgactCagctc	8,49E-07	26,31	HT-SELEX	loss
rs3777189	MAFB_1	pos	tttgagctCagt	0,0081786	pos	tttgagctGagt	4,60E-04	17,79	HT-SELEX	loss
rs3777189	MAFB_2	neg	agtgctgactGagctca	4,46E-06	pos	tgagctGagtcagcact	1,09E-07	40,77	HT-SELEX	loss
rs3777189	MAFK_1	pos	ttttgagctCag	0,017006	pos	ttttgagctGag	3,11E-04	54,66	HT-SELEX	loss
rs3777185	TFAP2C_3	pos	agccattcaggCa	3,78E-04	pos	agccattcaggTa	0,0055362	0,07	HT-SELEX	gain
rs3777185	TFAP2B_3	pos	agccattcaggCa	2,11E-04	pos	agccattcaggTa	0,0035692	0,06	HT-SELEX	gain
rs3777185	TCFAP2A_3	pos	agccattcaggCa	1,57E-04	pos	agccattcaggTa	0,0027967	0,06	HT-SELEX	gain
rs3777185	TFAP2A_6	pos	agccattcaggCa	2,56E-04	pos	agccattcaggTa	0,0041316	0,06	HT-SELEX	gain
rs3777185	TFAP2A_3	pos	agccattcaggCa	1,65E-04	pos	agccattcaggTa	0,0025362	0,07	HT-SELEX	gain
rs3777185	TFAP2C_5	neg	tGcctgaatggct	1,01E-04	neg	tAcctgaatggct	0,0023006	0,04	HT-SELEX	gain
rs4563648	SOX8_3	pos	tacaaatgtcaggGtga	3,78E-04	pos	tacaaatgtcaggAtga	0,0030834	0,12	HT-SELEX	gain

rs4563648	SOX21_1	pos	acaaatgtcaggGtg	2,01E-04	pos	acaaatgtcaggAtg	0,0019873	0,10	HT-SELEX	gain

rs4563648	TBR1_1	pos	ggGtgagaaa	1,29E-04	pos	ggAtgagaaa	0,0045558	0,03	HT-SELEX	gain
rs4563648	TBR1_2	pos	aggGtgagaaa	9,64E-05	pos	aggAtgagaaa	0,0032369	0,03	HT-SELEX	gain
rs4563648	TBX2_2	pos	aggGtgagaaa	1,01E-04	pos	aggAtgagaaa	0,0061029	0,02	HT-SELEX	gain
rs4563648	TBX21_5	pos	aggGtgagaa	1,35E-04	pos	aggAtgagaa	0,0052718	0,03	HT-SELEX	gain
rs4563648	SOX10_1	pos	acaaatgtcaggGtg	3,96E-04	pos	acaaatgtcaggAtg	0,0023008	0,17	HT-SELEX	gain
rs4563648	SOX2_4	neg	tcaCcctgacatttgta	3,60E-04	neg	tcaTcctgacatttgta	0,0027968	0,13	HT-SELEX	gain
rs4563648	TBX20_3	pos	gtcaggGtgagaaag	1,12E-04	pos	gtcaggAtgagaaag	0,0085897	0,01	HT-SELEX	gain
rs4563648	TBX20_5	pos	aaatgtcaggGtgaga	9,65E-05	pos	aaatgtcaggAtgaga	8,67E-04	0,11	HT-SELEX	gain
rs4563648	TBX20_1	pos	aaatgtcaggGtgaga	9,65E-05	pos	aaatgtcaggAtgaga	0,0015573	0,06	HT-SELEX	gain
rs4563648	TBX20_4	pos	aggGtgagaaa	3,11E-04	pos	aggAtgagaaa	0,0074198	0,04	HT-SELEX	gain
rs4563648	RARA_2	pos	gtggtacaaatgtcaggG	2,21E-04	pos	gtggtacaaatgtcaggA	0,00122	0,18	HT-SELEX	gain
rs4563648	SOX2_1	pos	tacaaatgtcaggGtga	3,97E-04	pos	tacaaatgtcaggAtga	0,0039352	0,10	HT-SELEX	gain
rs4563648	EOMES_1	pos	aggGtgagaaagt	1,43E-04	pos	aggAtgagaaagt	0,0047834	0,03	HT-SELEX	gain
rs4563648	TBX21_2	pos	aggGtgagaa	2,00E-04	pos	aggAtgagaa	0,00902	0,02	HT-SELEX	gain
rs4563648	SOX15_1	neg	caCcctgacatttgt	3,17E-04	neg	caTcctgacatttgt	0,0022996	0,14	HT-SELEX	gain
rs4563648	SPIC_1	pos	aggGtgagaaagtt	0,0074195	pos	aggAtgagaaagtt	3,43E-04	21,62	HT-SELEX	loss
rs3777189	NFE2L2.p2	pos	gagctCagtcagc	1,65E-04	pos	gagctGagtcagc	4,45E-06	37,05	SwissRegulon	loss
rs3777189	BACH2.p2	pos	gagctCagtcagcac	3,43E-04	pos	gagctGagtcagcac	8,41E-06	40,78	SwissRegulon	loss
rs3777189	ATF4.p2	neg	gctgactGagct	0,0037468	neg	gctgactCagct	3,60E-04	10,40	SwissRegulon	loss
rs3777189	PAX8.p2	neg	acagtgctgactGagctc	1,06E-04	neg	acagtgctgactCagctc	0,0039351	0,03	SwissRegulon	gain
rs3777189	FOS_FOS{B,L1}_JUN{B,D}.p2	pos	ctCagtca	0,0022958	pos	ctGagtca	1,29E-04	17,73	SwissRegulon	loss
rs3777189	FOSL2.p2	neg	tgactGagctca	0,0014828	neg	tgactCagctca	9,65E-05	15,37	SwissRegulon	loss
rs3777189	FOXP3.p2	neg	acagtgctgactGagct	0,0019871	neg	acagtgctgactCagct	2,21E-04	8,99	SwissRegulon	loss
rs3777189	NFE2.p2	neg	tgctgactGag	2,82E-04	neg	tgctgactCag	7,15E-06	39,46	SwissRegulon	loss
rs3777185	PAX6.p2	pos	ttcaggCagagttt	3,60E-04	pos	ttcaggTagagttt	0,0033985	0,11	SwissRegulon	gain
rs3777185	EN1,2.p2	pos	aggCagagttt	0,0055356	pos	aggTagagttt	2,96E-04	18,68	SwissRegulon	loss
rs4563648	RBPJ.p2	pos	ggGtgagaaagtttct	3,27E-04	pos	ggAtgagaaagtttct	0,0025366	0,13	SwissRegulon	gain
rs4563648	POU5F1_SOX2{dimer}.p2	neg	ctttctcaCcctgac	0,0047824	neg	ctttctcaTcctgac	3,97E-04	12,04	SwissRegulon	loss
rs4563648	NR1H4.p2	pos	caggGtgagaaagt	1,29E-04	pos	caggAtgagaaagt	0,0012202	0,11	SwissRegulon	gain
rs4563648	NR6A1.p2	pos	acaaatgtcaggGtgaga	2,56E-04	pos	acaaatgtcaggAtgaga	0,0012811	0,20	SwissRegulon	gain
rs3777189	TBX20_Heart-Tbx20-ChIP-Seq	neg	agtgctgactGa	2,32E-04	neg	agtgctgactCa	0,0013449	0,17	HOMER	gain
rs3777189	NFE2_K562-NFE2-ChIP-Seq	neg	gctgactGagct	8,34E-05	pos	gctGagtcagca	3,13E-06	26,65	HOMER	loss
rs3777189	JUN_motifA;K562-cJun-ChIP-Se	neg	gctgactGagct	9,56E-04	pos	gctGagtcagca	2,02E-05	47,26	HOMER	loss
rs3777185	NFIC_LNCAP-NF1-ChIP-Seq	neg	tctgaaactctGcctg	3,97E-04	pos	tcaggTagagtttcag	0,0043382	0,09	HOMER	gain
rs4563648	VDR_GM10855-VDR+vitD-ChIP	pos	caggGtgagaaagtttctaa	5,64E-05	pos	caggAtgagaaagtttctaa	6,16E-04	0,09	HOMER	gain
rs4563648	SPI1_Bcell-PU.1-ChIP-Seq	pos	aggGtgagaaag	0,0027965	pos	aggAtgagaaag	9,19E-05	30,43	HOMER	loss
rs4563648	RARG_ES-RARg-ChIP-Seq	pos	atgtcaggGtga	1,50E-04	pos	atgtcaggAtga	0,0022999	0,07	HOMER	gain

rs4563648	EOMES_H9-Eomes-ChIP-Seq	pos	ggGtgagaaa	1,12E-04	pos	ggAtgagaaa	0,0067295	0,02	HOMER	gain

Supplementary Table 5

Expression levels in CD138$^+$ MM plasma cells of transcription factors predicted to gain or lose a motifs as a result of variation at rs3777185, rs3777189 or rs4563648. Expression levels were calculated as FPKM values based on our RNA sequencing data. The table shows transcription factors with FPKM > 5. The table also shows the Pearson correlation between transcription factor and *ELL2* expression, as well as the predicted effect of the risk variants in terms of gain or loss of sequence motif.

**Gene symbol**	**Mean FPKM**	**Correlation with *ELL2***	**Predicted effect of risk variant**		
			**rs3777189**	**rs3777185**	**rs4563648**
JUN	1078,0	-0,254	loss		
ATF4	1041,0	0,096	loss		
FOS	949,1	-0,318	loss		
JUND	873,8	-0,259	loss		
FOSB	610,2	-0,396	loss		
JUNB	516,1	-0,063	loss		
IRF4	161,0	0,074			loss
STAT1	86,8	0,124			loss
NR1H2	65,8	-0,060			gain
BACH1	54,5	0,366	loss		
STAT2	37,7	-0,129			loss
MAFF	37,3	0,064	loss		
SPI1	26,3	-0,064			loss
FOSL2	18,2	0,003	loss		
MAFK	18,0	0,254	loss		
RARA	17,4	0,092			gain
ZEB1	13,0	0,578		loss	
MAFG	13,0	0,404	loss		
VDR	12,8	-0,065			gain
ETS1	11,9	-0,184			loss
KLF5	9,7	0,213			gain

Supplementary Table 6

Gene sets enriched (P <0.0001) for correlations with *ELL2* expression.

P-value	Direction	Gene set
0.000003	0.98	structural_constituent_of_ribosome
0.000003	0.99	ribosome
0.000003	-1	KRAB_box_transcription_factor
0.000003	0.99	Ribosomal_protein
0.000004	1	protein_biosynthesis
0.000004	1	Protein_biosynthesis
0.000004	0.96	RPGs
0.000007	1	small_GTPase_mediated_signal_transduction
0.000012	-1	Other_transporter
0.000013	1	translation_initiation_factor_activity
0.000014	1	Small_GTPase
0.000015	-1	Small_molecule_transport
0.000023	-1	Mitochondrial_carrier_protein
0.000028	1	regulation_of_translational_initiation
0.000033	-1	transport
0.000035	1	SEC
0.000049	0.97	cytosolic_large_ribosomal_subunit_(sensu_Eukaryota)
0.000076	1	regulation_of_translation
0.000084	1	Circadian_clock_system

Supplementary Table 7

Gene sets enriched (*P* <0.0001) for correlations with the *ELL2* genotype.

Negative direction value indicates enrichment for correlation with the risk allele.

Positive direction value indicates enrichment for correlation with protective allele.

**P-value**	**Direction**	**Gene set**
0.00000	-0.93	RPGs
0.00001	-0.74	structural_constituent_of_ribosome
0.00001	-1.00	KRAB_box_transcription_factor
0.00001	-0.51	protein_biosynthesis
0.00001	1.00	Cell_motility
0.00003	-0.82	ribosome
0.00003	-0.77	Ribosomal_protein
0.00005	0.97	oxidoreductase_activity
0.00009	1.00	glycolysis
0.00009	-1.00	mRNA_splicing_factor

Supplementary Table 8

Gene sets enriched (P <0.0001) for up- or downregulation in *ELL2* CRISPR-Cas9 knock-out versus wildtype L363 plasma cell leukemia cells.

P-value	Direction	Gene set
0.000003	1	structural_constituent_of_ribosome
0.000004	0.99	oxidoreductase_activity
0.000004	0.93	protein_biosynthesis
0.000004	1	Ribosomal_protein
0.000004	1	RPGs
0.000005	1	lysosome
0.000005	1	Protein_biosynthesis
0.000007	0.99	ribosome
0.000021	1	Electron_transport
0.000021	-1	protein_serine_threonine_kinase_activity
0.000028	1	Dehydrogenase
0.00003	-1	ubiquitin_cycle
0.000033	1	cytosolic_large_ribosomal_subunit_(sensu_Eukaryota)
0.000043	1	electron_transport
0.000056	-0.98	KRAB_box_transcription_factor
0.000056	1	endoplasmic_reticulum
0.000057	1	Amino_acid_catabolism
0.000061	-1	Other_transcription_factor
0.00007	0.95	integral_to_plasma_membrane

Supplementary Table 9

Gene sets enriched for correlations with ELL2 genotype in the mRNA sequencing data, and for change in expression by CRISPR-Cas9 ELL2 knock-out in the L363 cells. As shown, the only gene sets showing enrichment in the same direction of reduced ELL2 function were ribosomal gene sets.

Gene set	P_MMPC_rs3815768	Direction	P_L363	Direction
RPGs	0.000004	0.93	0.000004	1.00
Ribosomal_protein	0.000032	0.77	0.000004	1.00
protein_biosynthesis	0.000007	0.51	0.000004	0.93
ribosome	0.000028	0.82	0.000007	0.99
structural_constituent_of_ribosome	0.000006	0.74	0.000003	1.00
