# Supplementary material for: The multiple myeloma risk allele at 5q15 lowers ELL2 expression and increases ribosomal gene expression
Source: Nat Commun. 2018 Apr 25;9:1649. doi: 10.1038/s41467-018-04082-2 (PMC5917026; doi:10.1038/s41467-018-04082-2)
Supplement: Supplementary file 3 — Description of Additional Supplementary Files [file 41467_2018_4082_MOESM3_ESM.pdf]

Description of Additional Supplementary Files

File Name: Supplementary Data 1
Description: Pearson correlations between ELL2 expression and expression of other genes expressed in MMPCs (FPKM>5) in the Swedish-Norwegian RNA-sequencing data set.

File Name: Supplementary Data 2
Description: Pearson correlations between the ELL2 genotype and the expression of other genes expressed in MMPCs (FPKM<5) in the Swedish-Norwegian RNA-sequencing data set. For consistency with the plotting direction in Fig. 4b, the risk/low-expressing allele was encoded as 0 and the protective/high-expressing allele was encoded as 1 in this analysis. Hence, the genes at the top of the list correlate with protective allele, and the genes at the bottom of the list with the risk allele.
